# Molecular-dynamics simulation methods for macromolecular crystallography

**DOI:** 10.1107/S2059798322011871

**Published:** 2023-01-01

**Authors:** David C. Wych, Phillip C. Aoto, Lily Vu, Alexander M. Wolff, David L. Mobley, James S. Fraser, Susan S. Taylor, Michael E. Wall

**Affiliations:** a Computer, Computational and Statistical Sciences Division, Los Alamos National Laboratory, Los Alamos, NM 87545, USA; b Center for Nonlinear Studies, Los Alamos National Laboratory, Los Alamos, NM 87545, USA; c Department of Pharmaceutical Sciences, University of California, Irvine, Irvine, CA 92697, USA; d Department of Pharmacology, University of California, San Diego, La Jolla, CA 92093, USA; e Department of Bioengineering and Therapeutic Sciences, University of California, San Francisco, San Francisco, CA 94158, USA; f Department of Chemistry, University of California, Irvine, Irvine, CA 92697, USA; g Department of Chemistry and Biochemistry, University of California, San Diego, La Jolla, CA 92093, USA; Institut Laue-Langevin, Grenoble, France

**Keywords:** molecular-dynamics simulations, water structure, conformational ensembles, protein kinases

## Abstract

It is investigated whether molecular-dynamics (MD) simulations can be used to enhance macromolecular crystallography (MX) studies. Historically, protein crystal structures have been described using a single set of atomic coordinates. Because conformational variation is important for protein function, researchers now often build models that contain multiple structures. Methods for building such models can fail, however, in regions where the crystallographic density is difficult to interpret, for example at the protein–solvent interface. To address this limitation, a set of MD–MX methods that combine MD simulations of protein crystals with conventional modeling and refinement tools have been developed. In an application to a cyclic adenosine monophosphate-dependent protein kinase at room temperature, the procedure improved the interpretation of ambiguous density, yielding an alternative water model and a revised protein model including multiple conformations. The revised model provides mechanistic insights into the catalytic and regulatory interactions of the enzyme. The same methods may be used in other MX studies to seek mechanistic insights.

## Abstract 2

Atomistic simulations enhance protein crystallography, yielding mechanistic insights into a protein kinase involved in the regulation of fundamental biological processes that include metabolism, development, memory and immune response.

## 1. Introduction

Macromolecular crystallography (MX) has produced most of what is known about the atomic structure of proteins (Berman *et al.*, 2000 ▸). Historically, protein crystal structures have consisted of a single set of atomic coordinates (mean positions), temperature factors (positional variance) and occupancies (the fraction of the crystal where the atom is present). Structural models with just a single set of parameters for each atom, however, are limited in their ability to describe the full range of conformational variations that may be present in protein crystals. A number of approaches have been developed over the years to overcome this limitation, for example allowing crystal structure models with multiple conformations of selected side chains (Keedy *et al.*, 2015 ▸; Riley *et al.*, 2021 ▸) or ensemble models with multiple copies of the entire protein (Kuriyan *et al.*, 1991 ▸; Wall *et al.*, 1997 ▸; Burnley *et al.*, 2012 ▸; Ploscariu *et al.*, 2021 ▸). The resulting improvements in the descriptions of conformational heterogeneity are needed to understand the biological mechanisms involved in catalysis, molecular recognition and allostery (van den Bedem & Fraser, 2015 ▸).

Although multi-conformer modeling has improved the ability of protein crystallography to describe conformational heterogeneity, these methods can fail in regions where the crystallo­graphic density is difficult to interpret, as can occur, for example, at the protein–solvent interface. At the interface, protein atoms might be built into a region that is mainly occupied by solvent in the actual crystal. Similarly, standard methods for building ordered waters into a crystal structure (for example water picking; Adams *et al.*, 2010 ▸) make use of a supplied protein model; if this model contains errors then these methods can place waters into a region that is mainly occupied by protein. Addressing these issues might increase crystal structure accuracy; in particular, improving the water structure has been highlighted as a route to improved model accuracy (Holton *et al.*, 2014 ▸). Improved water structure models also can improve the modeling of molecular recog­nition and ligand binding (Baron *et al.*, 2010 ▸; Darby *et al.*, 2019 ▸), and yield insights into the regulation of protein dynamics and allostery (Leitner *et al.*, 2020 ▸) and the formation of protein–protein binding interfaces (Wong *et al.*, 2009 ▸). The hydration layer at the surface of proteins has been shown to be central to an understanding of protein structure, dynamics and function (Zhang *et al.*, 2007 ▸).

Because crystallographic density does not come with labels, independent information is sometimes required to help to distinguish the protein and solvent components. Recent developments suggest that molecular-dynamics (MD) simulations might be a promising means of obtaining such information. MD is a powerful computational method for providing insight into biomolecular structure and dynamics. In MD, a set of coordinates and potential energy parameters are used to model the atoms in a molecular system. The dynamics of the system are computed using Newtonian numerical integration, resulting in a trajectory that can be analyzed to capture phenomena on a time scale and at a resolution that is often inaccessible by laboratory measurements. Techniques borrowed from MD have been used for many years to sample conformations in crystallographic refinement (Adams *et al.*, 2010 ▸; Burnley *et al.*, 2012 ▸; Ploscariu *et al.*, 2021 ▸). In addition, crystalline MD simulation methods have advanced substantially in recent years, in a large part thanks to MD studies of diffuse X-ray scattering (Clarage *et al.*, 1995 ▸; Faure *et al.*, 1994 ▸; Héry *et al.*, 1998 ▸; Meinhold & Smith, 2005*a* ▸,*b* ▸, 2007 ▸; Wall *et al.*, 2014 ▸; Wall, 2018 ▸; Wych *et al.*, 2019 ▸; Meisburger *et al.*, 2020 ▸). Recent studies have shown that crystalline MD simulations can reproduce both the positions of ordered waters (Wall *et al.*, 2019 ▸) and the *B* factors from crystallographic refinement (Janowski *et al.*, 2016 ▸; Wall, 2018 ▸). These advances suggest that crystalline MD techniques have developed to the point where they might benefit macromolecular crystallography workflows.

Here, we investigate the utility of MD simulations in interpreting crystallographic density. Our MD approach is grounded in methods developed in a study that investigated the ability of MD simulations to recover crystallographic water structure (Wall *et al.*, 2019 ▸). In that study, it was found that crystalline MD simulations of endoglucanase were able to reproduce the positions of nearly all of the ordered waters from combined neutron and X-ray crystallographic experiments, but only when the protein heavy atoms were harmonically restrained to the crystal structure. The restraints biased the protein atoms towards the coordinates from single-structure experimental refinement and decreased the dynamics associated with anharmonic motions and structural heterogeneity. This left open the question of whether applying restraints to an ensemble, rather than a single structure, might improve the simultaneous modeling of protein conformational heterogeneity and the water network. To begin to address this question, we have developed an MD–MX procedure that uses ensemble-restrained MD simulations to revise the protein and water model in MX structures.

The system that we studied in developing this procedure is the catalytic subunit of mouse protein kinase A (PKA), a cyclic adenosine monophosphate (cAMP)-dependent protein kinase involved in the regulation of fundamental biological processes that include metabolism, development, memory and immune response. PKA exists in the cell as a tetramer consisting of two heterodimers, with each dimer containing a catalytic (PKA-C) subunit and a regulatory (PKA-R) subunit. PKA-C (the kinase domain) is activated by phosphorylation on its activation loop by PDK1 or via autophosphorylation as it comes off the ribosome (Keshwani *et al.*, 2012 ▸). In addition, because intracellular concentrations of ATP are high (millimolar) relative to ADP (∼10^3^-fold) and PKA (∼10^6^-fold), in cells the active ATP-bound form of PKA-C would be highly favored and the enzyme would be constitutively signaling. To prevent this, PKA-R binds to the active-site groove of PKA-C, blocking activity and the phosphorylation of partner proteins, and making PKA activity dependent on the second messenger 3′,5′-cyclic adenosine monophosphate (cAMP). Binding of cAMP to PKA-R releases it from PKA-C, unleashing the catalytic activity. PKA-C has been extensively studied using crystallography, neutron diffraction (Gerlits *et al.*, 2019 ▸), nuclear magnetic resonance (Masterson *et al.*, 2010 ▸) and other methods, due to its biological significance and its role as a prototypical kinase (Taylor *et al.*, 2004 ▸). There is also evidence of biologically important dynamics and allostery, making it a prime choice for study using crystalline MD (Masterson *et al.*, 2010 ▸). In the crystals used for our study, PKA-C is in complex with a small (20-amino-acid) pseudo-substrate peptide (IP20) from the heat-stable protein kinase inhibitor PKI, which blocks the active site. PKI competes with PKA-R *in vivo*, binding to the inhibitor site in certain tissues and leading to nuclear export of PKA (Liu *et al.*, 2020 ▸). This short peptide sequence is sufficient to block the release of ADP from the active site (the rate-limiting step in catalysis and subsequent substrate peptide release) and facilitates the crystallization of PKA-C and study of the structure and activity of the protein (Madhusudan *et al.*, 1994 ▸).

Cryocooling crystals, although a common practice to reduce radiation damage, decreases dynamics and traps proteins in conformations that may not be representative of their true structural ensembles and interactions *in vivo* (Keedy *et al.*, 2014 ▸). Cryocooling also can change the structure of active-site ordered water networks and the flexibility of side chains (Stachowski *et al.*, 2022 ▸). To capture PKA-C dynamics, therefore, crystals were grown at room temperature and X-ray crystallographic diffraction data were collected at 12°C. Crystal structures were obtained using both single-structure and ensemble refinement. Most likely due to the room-temperature crystallization conditions, we found that adenosine triphosphate (ATP), which binds with high affinity (60 n*M*) and is always used to stabilize the PKA-C–PKI complex during purification and crystallization (Whitehouse & Walsh, 1983 ▸), had hydrolyzed, allowing us to capture the immediate products of hydrolysis: adenosine diphosphate (ADP) and inorganic phosphate. Such hydrolysis had not been observed previously when crystals were grown at 4°C and data were collected at cryogenic temperatures.

Our study of PKA-C structure and function guided the development of the MD–MX procedure. The procedure involves three methods: (i) *density comparison*, enabling direct comparison with experimental data for assessment of the consequences of alternative modeling choices, including protonation states, (ii) *water building*, which calculates separate structure factors for the protein and water components of the simulation and produces an alternative ordered water model to that generated by crystallographic refinement, and (iii) *protein remodeling*, which uses both the MD density and trajectory snapshots to improve the modeling of residues where the interpretation of the density is unclear.

Combining all of these methods yielded a revised structural model with implications for PKA biology. In producing this model, we found sensitivity of the conformation of His294 to its protonation state, yielding insight into the potential role of this residue in the modulation of substrate-binding affinity (Deminoff *et al.*, 2006 ▸). The revised model includes a different conformation of Lys217 and introduces a coordinated free phosphate nearby. It also includes multi-conformer states for (i) Lys213, including a conformation associated with binding to the regulatory domain, and (ii) the active-site catalytic base Asp166, including a conformation that appears to be associated with progression of the phosphotransfer reaction. Based on the benefits that were seen on applying these methods to PKA-C, we recommend the incorporation of our MD–MX procedure into MX studies to produce multi-conformer models and to decide among the ambiguous interpretations of electron density that inevitably occur as part of standard model refinement. The resulting models may yield biological insights beyond those currently provided by standard crystallographic modeling techniques.

## 2. Materials and methods

### 2.1. Experimental design

This study aimed to determine whether MD simulations of protein crystals can aid in the modeling and interpretation of X-ray crystallographic data. Towards this aim, we collected room-temperature X-ray diffraction data from a crystal of the catalytic subunit (C-subunit) of PKA. The data were processed with two different selections for the resolution cutoff during the merging and scaling step of data processing, producing 2.4 and 1.63 Å resolution data sets with, respectively, higher and lower multiplicity and signal to noise in the highest resolution shells. An MD simulation of crystalline PKA was prepared using an ensemble model refined against the 2.4 Å resolution data set and was used to develop MD–MX methods to compare electron densities, produce an alternative solvent model and revise the protein structure using multi-conformer modeling. The models were validated and were used to produce revised crystal structures at 2.4 and 1.63 Å resolution.

### 2.2. Crystallographic data collection and refinement details

The C-subunit of PKA was recombinantly expressed in *Escherichia coli* and purified as described previously (Gerlits *et al.*, 2019 ▸). An additional Mono S cation-exchange step was used to separate the phosphorylation states, with the peak corresponding to three phosphorylations taken for crystallization. The C-subunit was concentrated to 8 mg ml^−1^ in 0.02 *M* KH_2_PO_4_ pH 6.5, 0.15 *M* KCl, 0.001 *M* DTT, and a ternary complex with inhibitor and ATP was formed with a 1:20:10:10 molar ratio of C-subunit:Mg:ATP:IP20 and a ternary complex with inhibitor and ATP was formed with a 1:20:10:10 molar ratio of C-subunit:Mg:ATP:IP20. The complex was crystallized by hanging-drop vapor diffusion at 20°C under previously described conditions (Gerlits *et al.*, 2013 ▸) in 20% PEG 4000, 0.05 *M* MES pH 5.2, 0.05 *M* MgCl_2_, 0.005 *M* DTT.

Room-temperature (12°C) X-ray diffraction data were collected on beamline 8.3.1 at the Advanced Light Source (ALS). The crystal was mounted on a MiTeGen loop and sealed in a MicroRT capillary. Two sweeps of 181 1° oscillation frames were collected between translations along the crystal. The sweeps were processed independently with a resolution cutoff of 2.4 Å using *DIALS*/*xia*2 in *CCP*4*i*2 (Winter *et al.*, 2018 ▸; Potterton *et al.*, 2018 ▸). *BLEND* in *CCP*4*i* (Foadi *et al.*, 2013 ▸) was then used to remove frames with radiation damage and to merge and scale the data set, with two different selections for the resolution cutoff in *BLEND* that resulted in a 2.4 Å resolution data set and a 1.63 Å resolution data set (merging statistics are reported in Table 1 ▸). The 2.4 Å resolution cutoff was obtained using a CC_1/2_ threshold of 0.5 in the highest resolution shell in processing the individual unmerged sweeps; in this case, *BLEND* indicated that all frames could be preserved (the merging statistics in Table 1 ▸ for the complete data set including both sweeps differ from those of the individual unmerged sweeps and should be interpreted accordingly; Foadi *et al.*, 2013 ▸). The 1.63 Å resolution cutoff was obtained using a CC_1/2_ threshold of 0.3; in this case, *BLEND* indicated that 104 frames from the end of sweep 1 and 128 frames from the end of sweep 2 needed to be eliminated to preserve acceptable merging statistics (presumably due to radiation damage). The 2.4 Å resolution data set, containing higher multiplicity but lower resolution data, was used to develop the MD–MX procedure and all associated structural models. The 1.63 Å resolution data set, which contained more than three times as many data points but with about one third of the multiplicity, was used to validate the final MD-revised structure and for a final round of refinement before submitting our structure to the PDB.

To obtain the initial crystal structure, molecular replacement was performed using *Phaser* (McCoy *et al.*, 2007 ▸) with PDB entry 3fjq (Thompson *et al.*, 2009 ▸) as the search model. Refinement and model building were performed in *Phenix* (Afonine *et al.*, 2012 ▸) and *Coot* (Emsley *et al.*, 2010 ▸). The input structure for ensemble refinement was prepared using an initial single-structure refinement model with full occupancies for main-chain and inhibitor peptide atoms, refined occupancies for the phosphate, ADP and magnesium ions in the active site, and refined sites and *B* factors for all atoms, as well as ordered waters placed by water picking, producing a model (*S*) with *R*_work_ = 0.1557 and *R*_free_ = 0.1838. The final model had 148 waters. Due to a lack of supporting density, this model had 14 residues missing in the flexible N-terminus and atoms missing in residues Gln176, Glu248 and Lys254. The waters around the active site of this model were removed and an OMIT map was created, eliminating bulk-solvent correction, to create a polder map (Liebschner *et al.*, 2017 ▸), which represents the density due to the water atoms in the region that were not included in the model.

An ensemble structural model, *E*, was refined with *phenix.ensemble_refinement* (Burnley *et al.*, 2012 ▸) for use in the crystalline MD. The number of ensemble members was set to 32 (one for each protein in the supercell system) by setting ensemble_reduction to False and restricting number_of_acquisition_periods and pdbs_per_block to eight and four, respectively (8 × 4 = 32).

### 2.3. MD system preparation

Previous crystalline MD simulations used a single structure-refinement model for the initial coordinates for all proteins in the supercell. Here, we used a modified *E* model and seeded each protein in the supercell with a unique conformation from the ensemble. Because the *E* model had 14 residues missing in the flexible N-terminus, as well as residues 23–24 of the PKI peptide unmodeled due to poor density, to produce the model which was used to seed the crystalline MD supercell these residues were modeled from PDB entry 1cmk (Zheng *et al.*, 1993 ▸) into the 32-structure ensemble; the added residues had *B* factors set to 120 Å^2^ and were refined in *Phenix* using rigid-body and real-space refinement followed by geometry minimization. No steric clashes were observed for the propagated system, and no issues were encountered in energy minimization, solvation or equilibration.

After removing crystallographic waters, each of the structures from the ensemble was propagated to a different location in a 2 × 2 × 2 supercell according to the symmetry information provided by the CRYST1 record (using two custom Python modules, *pdbio.py* and *propagate.py*, available at https://github.com/lanl/lunus/scripts). The full system was parameterized using *tleap* in *AMBER* (from the *AmberTools* package version 20.14; Case *et al.*, 2021 ▸) using the AMBER14SB force field (Maier *et al.*, 2015 ▸) for the protein and magnesium ions and the phosaa10 parameter set (Homeyer *et al.*, 2006 ▸) for the phosphorylated serine (SEP) and tyrosine (TPO) residues. The active-site-bound ADP was parametrized with the frcmod and prep files found in the Bryce Laboratory *AMBER* parameter database for cofactors (Meagher *et al.*, 2003 ▸). The phosphate was assumed to be doubly protonated ([H_2_PO_4_]^−^) and was subjected to mp2/aug-cc-pvdz QM geometry optimization to determine the geometry of the doubly protonated state before being parametrized in *tleap* with the gaff2 force field. The phosphate was placed identically into each ensemble structure with the least-squares distance to the crystal structure phosphate molecule minimized and with H atoms oriented away from the active-site magnesium ions.

The protein model was prepared in two alternative protonation states. In one model every histidine was doubly protonated (HIP), while in the other model the protonation/tautomeric states were assigned based on data from neutron diffraction: His87, His158 and His260 remained doubly protonated (HIP), His62, His131, His142 and His294 were protonated on the ɛ N atom (HIE) and His68 was protonated on the δ N atom (HID) (Gerlits *et al.*, 2019 ▸). The system was initially solvated with TIP3 waters (using *solvate* in *GROMACS*; Abraham *et al.*, 2015 ▸) and neutralized with chloride ions (using *genion* in *GROMACS*). Additional solvent atoms were replaced with magnesium and chloride ions sufficient to mimic the crystallization buffer MgCl_2_ concentration of 0.05 *M*.

### 2.4. Solvation and equilibration

Standard MD equilibration takes place in the NPT ensemble, in which the box side lengths are allowed to fluctuate to maintain constant pressure. However, to minimize errors in computing mean structure factors, it is important for us to perform simulations with the periodic box side lengths fixed, *i.e.* in the NVT ensemble (Wall *et al.*, 2014 ▸). Upon initial solvation and equilibration, the system was dramatically under-pressurized (about −1000 bar). To bring it to atmospheric pressure, the system was subjected to iterative rounds of solvation, minimization (using the steepest-descent algorithm) and NVT equilibration (500 ps total, with restraints on all heavy atoms with the restraint constant equal to 200 kJ mol^−1^ nm^−2^) until the average measured pressure, plus or minus the standard error, was in the range −100 to 100 bar. The system was then simulated for 10 ns of restrained ‘pre­liminary production’ at the same restraint strength, to ensure that the system had relaxed sufficiently, before the production run of 100 ns.

### 2.5. Production simulation

Both the HIP protonation model and the model with protonation states based on neutron diffraction were simulated with position restraints on heavy atoms using a spring constant of 200 kJ mol^−1^ nm^−2^. In all simulations, the time step was 2 fs (with LINCS constraints on hydrogen bonds) with coordinates output every 2 ps. During the early portion of the trajectories, the r.m.s.d. of atom positions between the MD protein structure and the crystal structure decreased steadily under the influence of the restraints; a plateau was reached at about 60 ns, and the 90–100 ns portion of the production trajectory was used for density analysis. The trajectory was down-sampled to one frame every 10 ps and processed to keep the molecules whole and to account for periodic boundary corrections (using the *trjconv* method in *GROMACS* with flags -pbc mol and -pbc nojump). Additional details of the simulation parameters and .mdp files are available from the authors upon request.

### 2.6. MD density analysis

Mean structure-factor .mtz files were calculated from the final 10 ns analysis trajectories using *xtraj.py* (Wych *et al.*, 2019 ▸), which takes a *GROMACS* .xtc trajectory file and .pdb topology file (taken from the first frame of the simulation) as input. MD structure-factor files were calculated separately for (i) the full system, (ii) protein, (iii) water, (iv) Mg^2+^ and (v) Cl^−^ atoms. The *FFT* method from *CCP*4 (version 7.1; Winn *et al.*, 2011 ▸) was used to convert the structure factors to electron-density maps. The maps were placed on an absolute scale (e^−^ Å^−3^) using the unit-cell *F*_000 _and volume, which were calculated using *cctbx* (Grosse-Kunstleve *et al.*, 2002 ▸).

The MD water density was analyzed with *PEAKMAX* and *SFTOOLS* from *CCP*4 to produce a set of peak positions with peak heights above a threshold of 1 e^−^ Å^−3^, representing ordered water positions from the MD. The recall statistics for crystallographic waters were calculated by isolating the unique waters from the set produced by peak finding (atom indices for symmetry-related copies have the same atom number), calculating the distance between crystallographic and MD-predicted waters (modulo unit-cell periodicity) and determining the fraction of crystallographic waters that have an MD-predicted water within a specified cutoff distance.

### 2.7. MD snapshots

Custom Python scripts (*pdbio.py* and *reverse_propagate.py*) were used to analyze the final frame of each simulation and to compare the original crystallographic ensemble with the ensemble from the final frame of each MD simulation (available at https://github.com/lanl/lunus/scripts).

## 3. Results

### 3.1. MD–MX analysis procedure

**Figure 1:**
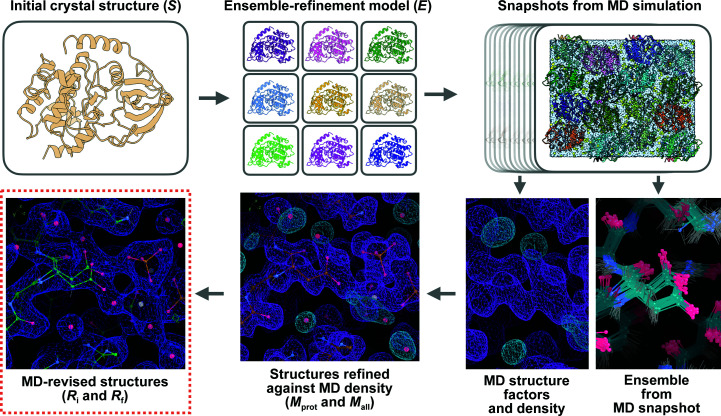
MD–MX procedure to revise macromolecular crystal structures. In this procedure, an initial crystal structure *S* (top left) is used to prepare an MD simulation model, using ensemble refinement to generate a set of diverse conformations (the ensemble-refinement model, *E*; top center). Snapshots from the MD simulation (top right) are used to generate simulated data and MD ensemble visualizations (bottom right; protein density in purple and water density in cyan). The initial structure is refined against the simulated data, leading to a revised protein structure, *M*_prot_, and a new water network, *M*_all_ (bottom center; densities on bottom right). The revised structure is refined against the experimental data to produce the initial MD-revised structure *R*_i_, and manual improvements are made guided by the MD density and snapshots, leading to a final MD-revised structure *R*_f_ (bottom left; 2*F*_o_ − *F*_c_ density in blue at 1σ with traces of positive 3σ *F*_o_ − *F*_c_ density visible in green).

Our main goal in this study was to determine whether crystalline MD simulations can provide insights into protein structure beyond those available using standard protein crystallography tools. To this end, we developed an MD–MX procedure for using crystalline MD simulations to revise crystal structures (Fig. 1 ▸). A distinguishing feature of this procedure is the calculation of structure factors and electron densities from crystalline MD trajectories. Such calculations previously enabled quantitative comparisons to assess the agreement of MD simulations with dynamics (Wall *et al.*, 2014 ▸; Wall, 2018 ▸; Wych *et al.*, 2019 ▸) and ordered waters (Wall *et al.*, 2019 ▸) in protein crystals. Here, we extend these capabilities to improve the modeling and interpretation of crystallographic data.

The MD–MX procedure was applied to crystalline PKA-C using X-ray data collected at a temperature of 12°C (Section 2). The data were processed in two different ways (Table 1 ▸): (i) using a 2.4 Å resolution cutoff, preserving all frames and yielding a lower resolution data set with a smaller number of unique reflections but with higher multiplicity and signal to noise, and (ii) using a 1.63 Å resolution cutoff, filtering out about 2/3 of the frames to achieve satisfactory merging statistics and yielding a higher resolution data set with more than three times as many unique reflections but with lower multiplicity and signal to noise, as the smaller number of frames led to fewer measurements per unique reflection. We used the 2.4 Å resolution data set to develop the MD–MX procedure and to produce the lower resolution PDB entry 7ujx. We used the 1.63 Å resolution data set for validation of PDB entry 7ujx against data not seen and for a final round of refinement to produce the higher resolution PDB entry 7v0g. Refinement statistics for these structures are given in Table 2 ▸.

The key steps of the MD–MX procedure are illustrated in Fig. 1 ▸. Firstly, an initial crystal structure (*S*) was obtained using molecular replacement and refinement adding ordered waters (*R* factors are given in Table 3 ▸). To generate an MD model with realistic conformational variation, the structure was further subjected to ensemble refinement, producing an ensemble-refinement model (*E*) with 32 protein structures (*R* factors are given in Table 3 ▸). These structures were randomly packed to assemble a crystalline system of 2 × 2 × 2 unit cells with four proteins per unit cell, and the resulting system was prepared for restrained MD simulations, in which the heavy-atom positions were biased toward the positions in *E* with harmonic restraints (Section 2).

After initial equilibration, the r.m.s.d. of the C^α^ coordinates between the MD system and structure *E* was about 0.436 Å (Supplementary Fig. S1). As the simulation progressed, the r.m.s.d. decreased and fell below 0.430 Å at about 60 ns, which is about where it remained until the 100 ns time point. We used the final 10 ns of the full 100 ns trajectory for analysis to ensure that the structure had relaxed sufficiently under the influence of the restraints. Snapshots were recorded every 10 ps (1000 total snapshots) and were used to produce ensemble visualizations and mean structure factors and densities from the full system, as well as mean structure factors and densities for the ion, the water, and the protein, inhibitor and active-site components separately.

The initial structure *S* was refined first against the MD structure factors computed only from the protein, inhibitor peptide and active-site components, producing structure *M*_prot_. Next, *M*_prot_ was refined against the total MD structure factors, including water and ion components, producing structure *M*_all_. The first-pass refinement against the protein structure factors allows the refinement to determine the positions of the protein atoms based on the MD data, avoiding the confusion of protein and solvent density. *M*_all_ was then refined against experimental data, producing an initial MD-revised structure *R*_i_. Lastly, information from the density and MD ensembles was used to remodel the structure by hand, yielding the final MD-revised structure *R*_f_.

Applying the MD–MX procedure to PKA-C produced the new information and insights presented here. The presentation is organized into sections, according to the method that was used to obtain the information. The following sections present the methods, along with the key results that they produced.

### 3.2. Density comparison to assess the agreement between MD and crystal structures

**Figure 2:**
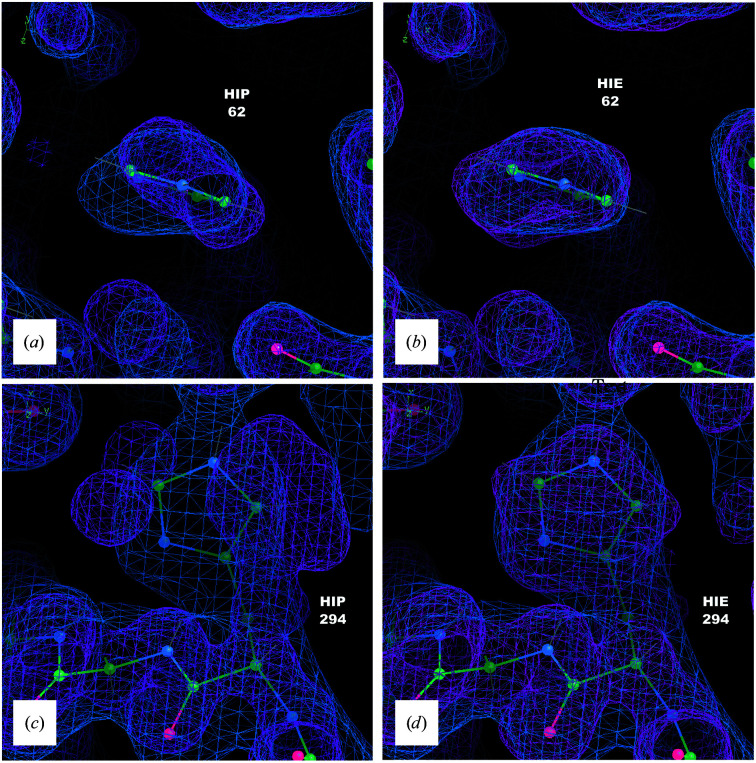
The His62 and His294 MD densities agree with the crystallographic density when the residues are protonated at the ɛ atom, but disagree when they are doubly protonated. Coordinates of *S* are shown as sticks with 2*F*_o_ − *F*_c_ density in blue (1σ isosurface) and the total density from a 90–100 ns segment of a 200 kJ mol^−1^ nm^−2^ MD simulation in pink (1σ isosurface). (*a*) His62: MD density from a doubly protonated (HIP) simulation. (*b*) His62: MD density from simulating with histidine singly protonated (on the ɛ N atom; HIE). (*c*) His294: MD density from a doubly protonated (HIP) simulation. (*d*) His294: MD density from simulating with histidine singly protonated (on the ɛ N atom; HIE).

The overlap between the MD density and the initial structure *S* is satisfactory for most of the protein. As an exception, two histidines (His62 and His294) showed changes in the density depending on their protonation states. The side-chain density for both agreed with *S* when singly protonated on the ɛ N atom, as in a neutron crystal structure (PDB entry 6e21; Gerlits *et al.*, 2019 ▸; Figs. 2 ▸ *b* and 2 ▸ *d*). When doubly protonated, however, the His62 imidazole ring rotates 45° and the His294 side chain enters a new rotameric state, which makes room for two ordered waters in the neighborhood (Figs. 2 ▸ *a* and 2 ▸ *c*).

To assess the agreement between the MD and crystallographic waters, MD densities were computed using just the water atoms. Peak-finding produced a picture of possible positions of ordered waters; to limit the number of peaks to those more likely to be significant, peaks where the density exceeds 1 e Å^−3^ were selected for comparisons with waters from the initial crystal structure (Section 2). Recovery of crystallographic waters was assessed using a recall statistic (the fraction of crystallographic waters that have an MD peak nearby): the simulation reproduces the positions of 137 of 148 (92.6%) of the crystallographic waters to within 1 Å, similar to a prior MD study of endoglucanase (Wall *et al.*, 2019 ▸).

### 3.3. Water building to generate an alternative solvent model

We used the water density to develop an alternative ordered water model for the crystal structure. To aid in the disambiguation of protein and solvent density, we performed ‘protein-first refinement’. The initial crystal structure *S*, stripped of its waters, was refined against the structure factors computed only from the nonsolvent (protein, inhibitor and active site) components of the simulation, producing *M*_prot_. Next, the *M*_prot_ model was used as an input for refinement against the structure factors computed from all atoms in the simulation, including the waters (Section 2). In this second refinement, waters were added using *Phenix*. The resulting model, *M*_all_, was used as an initial structure for refinement against experimental data. In this refinement, the waters in *M*_all_ were preserved and no new waters were added. The resulting initial MD-revised structure, *R*_i_, thus contains waters purely derived from structure factors computed from the MD simulation. The *R*_work_ and *R*_free_ values for *R*_i_ (0.1328 and 0.1777, respectively) decreased substantially compared with *S* (0.1557 and 0.1838, respectively) (rows 1 and 3 in Table 3 ▸). The *M*_all_ structure had 494 waters (compared with 148 from *S*). Most of these additional waters appear in the first and second hydration layers (Supplementary Fig. S2). The ordered water model in *M*_all_ reproduced 136 of the 148 (91.9%) waters in *S* to within 1 Å, which is very similar to the value observed using peak finding above (137 of 148).

### 3.4. Protein remodeling where the interpretation of density is unclear

The water-building method yielded a revised crystal structure *R*_i_ with an alternate water model and improved *R* factors compared with the initial crystal structure *S*. The *R*_i_ structure, however, still had regions that needed improvement. To address these issues, we developed the protein-remodeling method.

Protein remodeling uses MD density and ensemble snapshots to guide further revisions to the structure resulting from the water-building step (Fig. 1 ▸, bottom right). The MD density is used to determine where there might be an alternate conformation or a different way to build a side chain and to suggest where small molecules might be built into the model. The MD ensemble snapshots can suggest (multiple) alternate conformations for rebuilding the side chain as starting points for further rounds of crystallographic refinement. In applying these steps to the *R*_i_ structure, the revisions slightly improved the agreement with the data: the final MD-revised structure *R*_f_ had an *R*_work_ of 0.1282 and an *R*_free_ of 0.1765, compared with values of 0.1328 and 0.1777, respectively, for the *R*_i_ structure (Table 3 ▸). Importantly, the resulting structure *R*_f_ yielded new insights into the mechanisms of PKA activity and regulation (Section 4).

**Figure 3:**
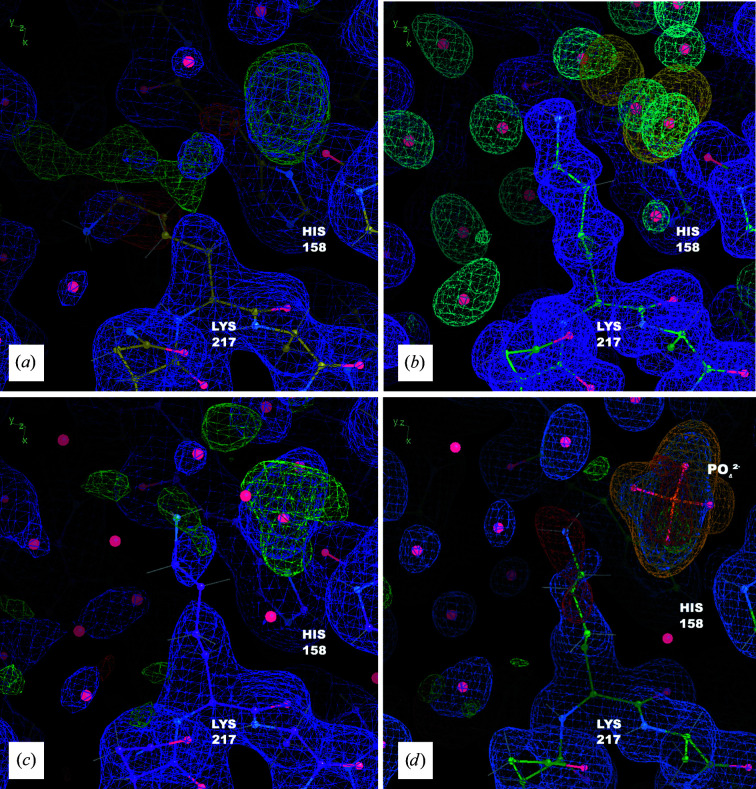
The MD–MX procedure guides remodeling of Lys217 and reveals a bound inorganic phosphate. (*a*) Coordinates, 2*F*_o_ − *F*_c_ density (blue, 1σ isosurface) and *F*_o_ − *F*_c_ density (positive in green and negative in red, 3σ isosurface) from *S*. (*b*) Coordinates from *M*_all_, with MD protein (purple, 1σ isosurface), solvent (blue, 3σ isosurface) and chloride density (yellow, 10σ isosurface), from the 90–100 ns segment of the simulation; the MD simulation suggests a different conformation for the side chain and a number of ordered waters; it also includes a spot of chloride density in the same position as the positive difference density in (*a*). (*c*) Coordinates, 2*F*_o_ − *F*_c_ density (blue, 1σ isosurface) and *F*_o_ − *F*_c_ density (positive in green and negative in red, 3σ isosurface) from *R*_i_; the shape of the difference density is suggestive of a coordinated free phosphate molecule. (*d*) Coordinates, 2*F*_o_ − *F*_c_ density (blue, 1σ isosurface) and *F*_o_ − *F*_c_ density (positive in green and negative in red, 3σ isosurface) from model *R*_f_ refined against the high-resolution data (PDB entry 7v0g): the revised side-chain conformation, water network and phosphate are plausible and improve the difference density in the region. His158 is also shown as a reference point. Polder OMIT map density for the phosphate is shown at a level of 5σ (orange) confirming that the addition of this molecule is reasonable.

**Figure 4:**
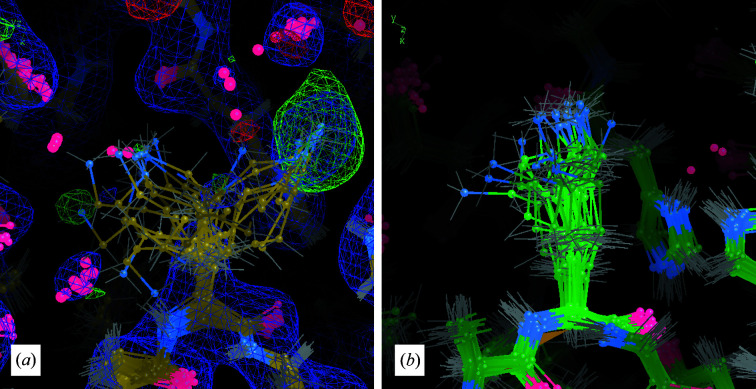
An MD snapshot guides remodeling of Lys217. (*a*) Coordinates, 2*F*_o_ − *F*_c_ density (blue, 1σ isosurface) and *F*_o_ − *F*_c_ density (positive in green, negative in red, 3σ isosurface) from the ensemble-refinement model *E*: the Lys217 amino group is diverse and includes extensions into off-backbone density and positive difference density where the phosphate was placed in the *R*_f_ structure (Fig. 3 ▸). (*b*) Final-frame MD snapshot: the side-chain conformations are more tightly clustered, extending straight from the backbone.

We first describe the revisions in the modeling of Lys217. In structure *S*, Lys217 shows negative difference density at the end of the side chain and positive difference density extending from the β C atom (Fig. 3 ▸ *a*). In the ensemble structure *E* the side-chain atoms have diverse conformations, some of which are close to those in structure *S*, but most of which are very different (Fig. 4 ▸ *a*). Many of the individual structures in *E* have the side chain positioned so that the amino group falls within a spot of density that is far from the side-chain atoms in *S* (Fig. 4 ▸ *a*).

The water-building step yielded a different side-chain conformation for Lys217. The 1σ MD density envelope for the protein extends perpendicular to the backbone, and the conformation of Lys217 in *M*_all_ was refined lying within this envelope (Fig. 3 ▸ *b*). The side-chain atoms in the *R*_i_ structure are consistent with this conformation, suggesting that it is supported by the experimental data (Fig. 3 ▸ *c*). The snapshot from the final frame of MD is consistent with the extended side-chain conformation, and there is less variation among the structures from the snapshot than there is in *E* (Fig. 4 ▸ *b*). None of the conformations in the snapshot are close to that in *S* and none are close to those that fall within the extra density occupied by the amino groups in *E*.

The protein-remodeling step provided a path forward for modeling the extra density near Lys217. Envelopes in the MD densities from both the isolated solvent and chloride ions overlapped the extra density, at first suggesting that it might include contributions from both (Fig. 3 ▸ *b*). However, adding a water or chloride ion to the structural model left a significant amount of positive difference density in the region after refinement against experimental data, indicating that more electrons were required (Fig. 3 ▸ *c*). This finding, along with the shape of the difference density, suggested the possibility that it might correspond to an inorganic phosphate. Although free phosphate was not included in the simulation, phosphate was present in the buffer, and at the experimental pH (<6.5) phosphates would be negatively charged, like the simulated chloride ions. We therefore placed a phosphate into the difference density. In the resulting structure *R*_f_ the phosphate density appears reasonable. To further validate the placement of the inorganic phosphate, we computed a polder OMIT map (Liebschner *et al.*, 2017 ▸) for the region near Lys217 using the higher resolution data. The shape of the density above 5σ provides clear evidence for the phosphate (Fig. 3 ▸ *d*).

**Figure 5:**
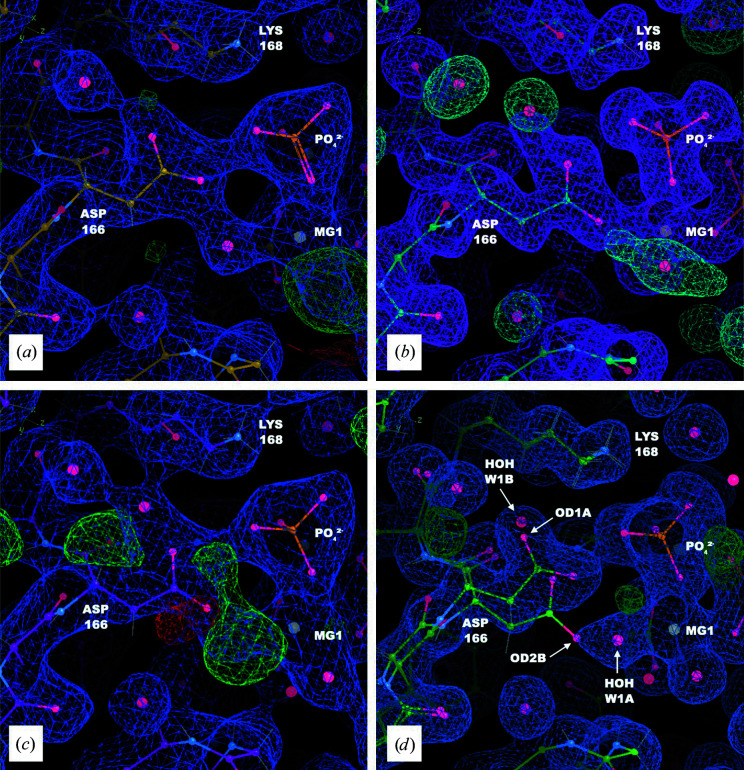
The MD–MX procedure yields a multi-conformer model of Asp166. (*a*) Coordinates, 2*F*_o_ − *F*_c_ density (blue, 1σ isosurface) and *F*_o_ − *F*_c_ density (positive in green and negative in red, 3σ isosurface) from model *S*. (*b*) Coordinates from the *M*_all_ model, with MD protein and cofactor density (purple, 1σ isosurface) and solvent density (blue, 3σ isosurface) from the 90–100 ns segment of the 200 kJ mol^−1^ nm^−2^ simulation. (*c*) Coordinates, 2*F*_o_ − *F*_c_ density (blue, 1σ isosurface) and *F*_o_ − *F*_c_ density (positive in green and negative in red, 3σ isosurface) from model *R*_i_. (*d*) Coordinates, 2*F*_o_ − *F*_c_ density (blue, 1σ isosurface) and *F*_o_ − *F*_c_ density (positive in green and negative in red, 3σ isosurface) from model *R*_f_ refined against the high-resolution data (PDB entry 7v0g): the water in chain W associated with Asp166 (labeled HOH W1A/B) is modeled as a multi-conformer atom, with the A conformer adjacent to the magnesium and the B conformer adjacent to OD1 on the A conformer of the side chain.

**Figure 6:**
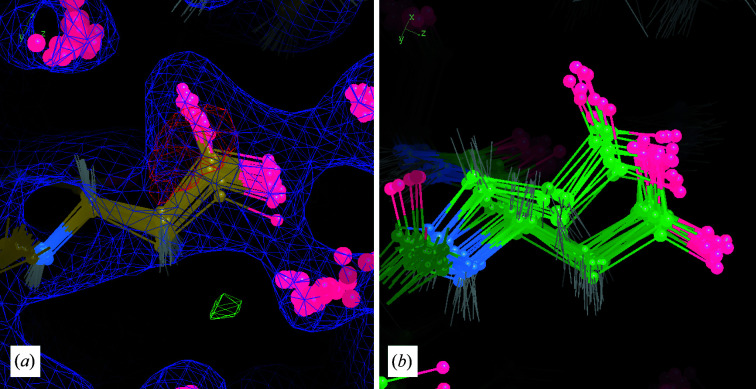
An MD snapshot guides the multi-conformer modeling of Asp166. (*a*) Coordinates from ensemble refinement against experimental data, with 2*F*_o_ − *F*_c_ density (blue, 1σ isosurface) and *F*_o_ − *F*_c_ density (positive in green, negative in red, 3σ isosurface) from ensemble refinement. (*b*) Coordinates from the reverse-propagated final frame of the 200 kJ mol^−1^ nm^−2^ crystalline MD simulation. The MD ensemble exhibits significantly more structural heterogeneity than the ensemble from refinement, with about half of the side chains in the A conformation and half in the B conformation.

Protein remodeling also yielded a multi-conformer model of Asp166, which serves as the catalytic base at the active site. There is substantial difference density in the active site near MG1 in *S* near Asp166 (Fig. 5 ▸ *c*). There is also some positive difference density on either side of the side chain of Asp166: next to one of the O atoms on the α-carboxylic acid and next to the C^β^—C^γ^ bond (Fig. 5 ▸ *a*). In *E*, Asp166 shows limited structural heterogeneity, with most of the side chains positioned as in *S* (Fig. 6 ▸ *a*). In the final-frame MD snapshot Asp166 is similar to *S* in about half of the structures; in the other half, however, the side chain is shifted towards MG1 (Fig. 6 ▸ *b*). The MD density also suggested a coordinated water adjacent to the O atom of the α-carboxylic acid and other waters next to MG1 (Fig. 5 ▸ *b*).

Based on the MD snapshot, we revised the model using alternate conformations for residues 163–169 (three residues to either side of Asp166 to accommodate the shift associated with the B conformation). At first it was not obvious how to model the water, because the MD water density appeared to clash with the multi-conformer model (Fig. 5 ▸ *c*). To resolve this issue, a single water molecule was modeled with alternate conformations on either side of Asp166. In the resulting structure (Fig. 5 ▸ *d*), the A position of HOH W1 is close to the B position of OD2 of Asp166 and the B position of HOH W1 is close to the A position of OD1 of Asp166; however, the clashes are avoided if the A and B conformations of Asp166 co-occur with their corresponding waters.

The revised model was refined against the crystallographic data, yielding *R*_f_. This structure was deposited in the PDB as entry 7ujx. In *R*_f_, the density near Asp166 is substantially improved compared with *R*_i_, with nearly all 3σ difference density eliminated, even when refined against the higher resolution data (Fig. 5 ▸ *d*). In addition, the occupancies of the alternate conformations for residues 163–169 are consistent with the water occupancies: the protein and water A conformation occupancies are 0.80 and 0.73, respectively, and the protein and water B conformation occupancies are 0.20 and 0.27, respectively.

**Figure 7:**
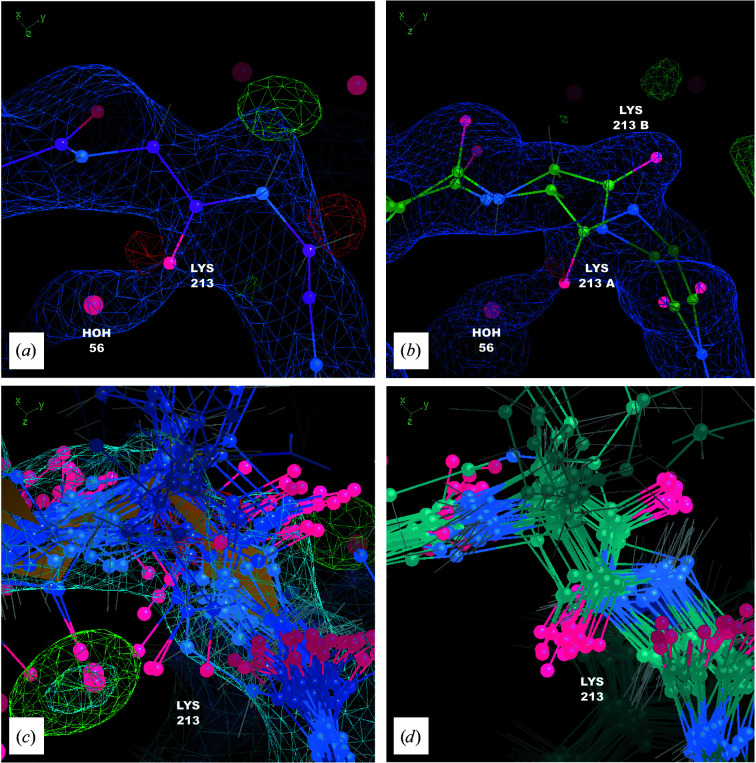
The MD–MX procedure yields a multi-conformer model of Lys213. (*a*) Initial MD-revised model (*R*_i_) coordinates (magenta), 2*F*_o_ − *F*_c_ density (blue, 1σ isosurface) and *F*_o_ − *F*_c_ density (positive in green, negative in red, 3σ isosurface). (*b*) Final MD-revised model (*R*_f_) coordinates (green), 2*F*_o_ − *F*_c_ density (blue, 1σ isosurface) and *F*_o_ − *F*_c_ density (positive in green, negative in red, 3σ isosurface) from refinement against the high-resolution data: the A conformation is at 46% occupancy, the B conformation at 54% occupancy and water 56 in chain S at 100% occupancy. (*c*) Coordinates from ensemble refinement model (*E*; blue), 2*F*_o_ − *F*_c_ density (blue, 1σ isosurface) and *F*_o_ − *F*_c_ density (positive in green, negative in red, 3σ isosurface). (*d*) Coordinates from the ensemble snapshot from MD simulation (turquoise): the ensemble snapshot suggests a clear multi-conformer state defined by a peptide flip.

Like Asp166, protein remodeling produced a multi-conformer model of Lys213. The initial MD-revised model (*R*_i_) showed negative difference density close to the backbone O atom of Lys213 and a large spot of positive difference density on the other side of the backbone (Fig. 7 ▸ *a*). There was also a close contact (1.97 Å) between the backbone O atom of Lys213 and a water O atom (HOH 56 in chain S) nearby. These features initially suggested the possibility of a peptide flip; however, substantial difference density remained after performing the flip in a single tt+ configuration (Touw *et al.*, 2015 ▸; not shown). An inspection of the final-frame MD snapshot revealed a clear multi-conformer state for Lys213, with about half of the structures (A conformation) in the original configuration and half (B conformation) in the tt+ configuration (Fig. 7 ▸ *d*). These states could not be identified clearly in *E*, which is much more disordered (Fig. 7 ▸ *c*). The model was revised to include a multi-conformer state for residues 212–214 (*R*_f_), with the B conformation taken from one of the MD snapshots, reducing the difference density (Fig. 7 ▸ *b*). The A conformation in structure *R*_f_ refined to an occupancy of 0.46 and the B conformation to an occupancy of 0.54; these values are consistent with what is seen in the MD snapshot.

To determine whether structure *R*_f_ was consistent with data not yet seen, we compared it with the 1.63 Å resolution data set, which includes a large number of data points beyond the 2.4 Å resolution data set used to refine *R*_f_ (Section 2). The initial *R* factors were *R*_work_ = 0.1871 and *R*_free_ = 0.1873 (the values are similar as the *R*_free_ flags had just been defined prior to the comparison). The same comparison for the initial structure *S* yielded substantially higher *R* factors: *R*_work_ = 0.1991 and *R*_free_ = 0.2058. After three macrocycles of refinement of *R*_f_ against the high-resolution data, the *R* factors decreased to *R*_work_ = 0.1563 and *R*_free_ = 0.1785, which are comparable to those obtained for the lower resolution data (Table 3 ▸). For comparison, subjecting the initial crystal structure *S* to the same refinement strategy against the high-resolution data yielded *R*_work_ = 0.1751 and *R*_free_ = 0.1981. An additional refinement step adding ordered waters yielded *R*_work_ = 0.1634 and *R*_free_ = 0.1841; these values are still higher than the values obtained after refining the *R*_f_ structure. Moreover, refinement against the higher resolution data yielded no significant changes in *R*_f_ for Asp166, Lys213, Lys217 or the water model and produced comparable difference density in these regions.

Further rounds of refinement were performed to improve the high-resolution model using the MD–MX procedure. The structure resulting from the refinement of *R*_f_ against the 1.63 Å resolution data set was refined first with isotropic *B* factors (three macrocycles) followed by refinement with anisotropic *B* factors (six macrocycles). Additional multi-conformer residues were added using the MD–MX procedure, based on combined inspection of the difference density, MD density and the ensemble from the final MD snapshot: Lys21, Glu24, Lys28, Glu31, Ser34, Gln35, Asn36, Gln39, Met71, Lys78, Lys81, Asn99, Val104, Lys105, Glu121, Ser130, Gln177, Lys192, Ile244, Gln245, Ser259, Ser263, Arg270, Lys285, Lys295, Glu311 and Glu331. Two additional water molecules were added manually while traversing the structure to identify multi-conformer residues (waters 495 and 496 in chain S). Finally, atoms with weak support in the density (high RSRZ) were removed in residues Lys16, Glu17, Ala20, Lys21, Gln242, Ile244, Glu248, Arg256, Lys285, Lys309, Glu333, Arg336 and His23 of the peptide (chain I). The structure was refined against the 1.63 Å resolution data set, yielding *R*_work_ = 0.1163 and *R*_free_ = 0.1625. This final higher resolution structure was deposited in the PDB as entry 7v0g. As there was difference density for this model associated with the coordinated phosphate near Lys217, a polder OMIT map (Liebschner *et al.*, 2017 ▸) was calculated, which confirms the presence of the phosphate (Fig. 3 ▸ *d*).

### 3.5. Some additional insights into the MD–MX pipeline

A key step in the MD–MX procedure is the refinement of structure *S* into the MD structure factors calculated from just the protein, inhibitor peptide and active-site atoms (‘protein-first’ refinement). To gain insight into the importance of this step, we altered the procedure by skipping it and instead directly refined *S* against the MD structure factors calculated from the entire system. The resulting model differed in three key ways from that resulting from protein-first refinement. (i) There are isolated cases where side chains are built into water density (for example, Lys28 and Lys295). As refinement against experimental data is always performed using the total structure factors, such errors might not be uncommon in published crystal structures. (ii) The *R* factors obtained after initial refinement of this structure against the experimental data (*R*_i_) are higher (*R*_work_ of 0.1382 rather than 0.1328 and *R*_free_ of 0.1795 rather than 0.1777), indicating that protein-first refinement improved the agreement with the data. (iii) The model contained about 50 fewer waters. Inspection of the missing waters revealed that they are primarily in the second hydration layer and beyond (not shown). The overall shape of the water density envelope appeared to be similar with or without protein-first refinement; the differences nevertheless influenced the water picking, perhaps during the filtering step.

To gain additional insight into how the MD–MX method for modeling structural heterogeneity compares with the picture obtained from ensemble refinement, we re-ran the ensemble refinement using the 1.63 Å resolution data set. In the resulting ensemble, many side-chain conformations of Lys217 were still modeled into the phosphate density, indicating that the higher resolution data did not help to distinguish protein from solvent in this region. Although the ensemble did not provide strong evidence for an alternate conformation for Asp166, it did contain some side-chain conformations consistent with the B conformation identified by the MD and showed substantial negative difference density associated with the side chain of Asp166. Finally, although the multiple peptide-plane conformations for Lys213 obtained using the MD–MX procedure were not identified using low-resolution ensemble refinement, high-resolution ensemble refinement did suggest two alternate conformations for the Lys213 peptide plane.

## 4. Discussion

Computing crystallographic densities is a key element of the MD–MX procedure, as it enables the simulations to be compared directly with diffraction data, provides a means of obtaining an alternative water model and provides information that can be used to remodel the protein structure, including multiple conformations. Density comparisons helped us to identify two residues whose conformations are especially sensitive to the protonation state: His62 and His294. This approach could be used in other systems and extended to investigate the impact of protonation on local ordered water structure and interactions with neighboring residues. Structural ensembles and calculated densities or structure factors from MD simulations may also prove to be useful for optimizing MD force fields using crystallographic data.

In addition to providing direct comparisons to the data, densities also provide a general approach to visualize structural ensembles that can complement other types of visualizations. For example, two sets of ensemble coordinates might appear to be similar but have different mean densities, as we saw in the case of His62. Similarly, two sets of ensemble coordinates may appear to be different while their average densities are very similar: a potential issue for ensemble refinement. For example, when there are too many structures, the ensemble coordinate view becomes too cluttered to be meaningful; in such cases, the density view provides a useful means of comparison. Indeed, a recent solution-state MD study of protein water rehydration used density computations to overcome difficulties in using atomic coordinates directly to assess whether binding sites of interest were occupied by waters (Ge *et al.*, 2022 ▸). Density comparisons such as those performed here therefore should prove useful in MD applications beyond crystallo­graphy.

The MD–MX solvent model includes many ordered waters that were not present in the initial crystal structure. The modifications to the water network appear to be plausible in local regions of the protein that were examined in detail. For example, we removed waters from the active site and calculated a polder OMIT map (Liebschner *et al.*, 2017 ▸) for the region; this density was extended and lacked strong peaks, which might help to explain why water picking did not place ordered waters in this region in the initial structure *S*. Nearly all of the MD waters around the active site were supported by polder density. Many of them were also near waters in a different structure obtained using neutron diffraction data (Gerlits *et al.*, 2019 ▸). At present, we cannot say how many of the MD waters outside the regions that we examined carefully should be treated with high confidence. In some cases, there are waters with relatively weak support from the density compared with what one is used to seeing in crystallography. There are even some isolated cases where the waters are associated with nearby negative difference density after refinement against experimental data; nevertheless, we chose to retain all MD waters because our aim was to assess a water model entirely determined by MD simulations and to determine what we can learn about the crystal structure from such a model.

The MD–MX procedure helped to mitigate some of the pitfalls of protein crystallography that result from the inability to distinguish between protein and solvent density. Both single-structure and ensemble refinement may model side chains into total density which would otherwise be ambiguous as to the conformation and/or identity of the side chain (protein, solvent or other molecules). The water-building and protein-remodeling methods provided information to resolve such ambiguities. The key information used in protein remodeling, beyond what is used in water building, comes from MD snapshots (Figs. 4 ▸ and 6 ▸). In particular, the MD snapshots allowed us to identify three places where the crystal structure could be improved in ways that were not suggested by ensemble refinement: Lys217, Asp166 and Lys213.

Lys217 provides a clear example of how MD snapshots enable the disambiguation of density. The protein-remodeling method provided a clear path to improving the side-chain model and enabled us to model a phosphate in this region. Even though the simulation is restrained to the diverse set of structures in model *E*, the conformation of Lys217 in the MD snapshot was relatively homogeneous and deviated substantially from the ensemble refinement (Fig. 4 ▸ *b*). Some of the atoms in the side chain of Lys217 moved by 5 Å or more, suggesting a force-field bias on these atoms that is stronger than a hydrogen bond (>25 kJ mol^−1^ at 200 kJ mol^−1^ nm^−2^). In such cases where the crystal structure is energetically unfavorable in the MD force field, the MD simulations can be especially useful in providing complementary information for protein crystallography. Lys217 is a striking example of this, as neither single-structure nor ensemble refinement against low- or high-resolution data suggested the presence of the phosphate, and the side-chain conformations modeled into the phosphate density by standard refinement techniques were heavily unfavored by the force field.

The phosphate near Lys217 was not observed in the previous joint X-ray and neutron diffraction data set, where there is a water molecule in the same position instead. However, in this experiment free phosphate was not present in the crystallization buffer, Sr^2+^ rather than Mg^2+^ was used to co-crystallize and, although ADP is present, the γ-phosphate of ATP is transferred to a serine on the substrate peptide (Gerlits *et al.*, 2019 ▸). It thus represents a product complex, whereas in our structure the γ-phosphate was transferred to water. The procedure followed in this example might be generalizable to other MD studies: charged ions in the solvent, which are often required for the neutralization or modeling of buffer salts in MD simulations, may serve as proxies for other charged molecules in the experimental buffer that are not included in the explicit solvent of the simulation.

In addition to guiding the remodeling of a single conformation of a side chain, as for Lys217, the cases of Asp166 and Lys213 (as well as the numerous cases added during the high-resolution refinement) show that the MD–MX procedure can help to develop multi-conformer models. Although both the single-structure and ensemble-refined models of Asp166 have difference density in the region of the side chain, neither suggests a path forward; in contrast, the MD snapshot clearly suggests a specific multi-conformer model (Fig. 6 ▸). In the case of Lys213, the MD–MX procedure at low resolution yielded a two-state model for the backbone that was only apparent at high resolution using ensemble refinement. These examples show that the MD–MX procedure can not only guide single-structure revisions, but can also be used to develop multi-conformer models of protein structure, including peptide flips (Croll, 2018 ▸; Touw *et al.*, 2015 ▸). In addition, these comparisons reveal that the MD–MX procedure can overcome limitations in standard methods for interpreting crystallographic data, even when the limitations persist after increasing the resolution of the data.

The PKA-C crystals studied here were prepared in a relatively uncommon state: with both crystal preparation and diffraction performed at room temperature. It is possible that the room-temperature preparation allowed us to capture an intermediate catalytic state with ATP hydrolyzed to ADP and with the free phosphate and two magnesium ions bound in the active site. The active site has previously been observed with ATP hydrolyzed in a mutant (Madhusudan *et al.*, 2002 ▸). A neutron diffraction structure (Gerlits *et al.*, 2019 ▸) captured the active site in a similar state, but with the phosphate transferred to a residue on a substrate peptide. The present structure is unique in that free phosphate is present in the active site. In this structure we have thus captured the slow ATPase activity of the C-subunit which leads to a reduced affinity for the inhibitor peptide (Zimmermann *et al.*, 2008 ▸).

The sensitivity of the conformation of His294 to the protonation state was notable: when doubly protonated, the side chain of His294 entered an entirely different rotameric state, making room for two ordered waters to enter the space occupied by the side chain when singly protonated. This sensitivity might be biologically significant, since previous studies have shown that this region, and this residue in particular, plays a role as a distant tethering site for the release of the protein substrate, with mutations of residues in this region modulating the affinity of binding (Deminoff *et al.*, 2006 ▸, 2009 ▸). His294 might become doubly protonated when the molecule is brought close to charged groups; for example, the regulatory subunit PKA-RII binds to the membrane, and when bound would position PKA-C such that this region would be near phospholipid head groups (Zhang *et al.*, 2015 ▸; Lu *et al.*, 2020 ▸).

**Figure 8:**
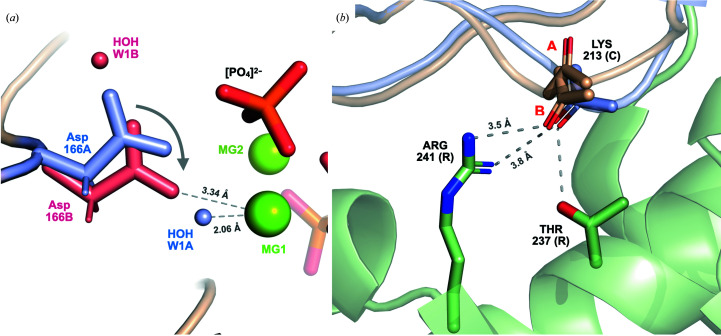
Implications of the MD-revised structure for PKA-C mechanisms. (*a*) The alternative conformation of Asp166 suggests a mechanism for the progression of catalysis. In the A conformation (blue), Asp166A is shifted away from MG1 and water O atom HOH W1A is coordinated to MG1. In the B conformation (red), Asp166B is shifted down towards MG1 and makes room for water O atom HOH W1 to occupy a different site above the side chain (where it would have clashed with conformation A of Asp166). The distance between OD2 of Asp166B and MG1 is larger (3.34 Å) than the distance between the O atom of water HOH 1A in chain W and MG1 (2.06 Å), potentially weakening coordination to MG1 and encouraging its escape from the active site post-catalysis. The multiple conformations suggest the possibility of a concerted motion (arrow) for Asp166 associated with progression of the phosphotransfer reaction. (*b*) The alternative conformation of Lys213 is consistent with the backbone pose for binding to the regulatory subunit. The final MD-revised structure (*R*_f_, light brown) models Lys213 of the catalytic subunit (PKA-C) with two conformers defined by a peptide flip (side chain not shown). The B conformer of Lys213 is consistent with the backbone O-atom orientation of PKA-C bound to the regulatory subunit RIα (PDB entry 2qcs, with the catalytic subunit shown in light blue and the regulatory subunit shown in light green). The backbone O atom of Lys213(C) is close to Thr237 and Arg241(R).

Previous studies have shown that both Lys213 and Lys217 are crucial for binding to the regulatory subunit, with charged-to-Ala mutations for both residues having a significant effect on binding affinity to RIα (Gibson *et al.*, 1997 ▸). The extended conformation of Lys217 and the addition of inorganic phosphate in this region may have implications for binding to the regulatory subunit. At present, we are not aware of any biological role that this phosphate may play; it would be interesting to determine whether it might have a role in regulating activity. The region surrounding Lys213 is a basic surface (PRS2) involved in the binding to PKA-R. In PDB entry 2qcs (Gibson *et al.*, 1997 ▸), Lys213 makes a crucial link to Thr237 and Arg241 of RIα (Fig. 8 ▸ *a*), which together form an allosteric hotspot that coordinates communications between two cyclic nucleotide-binding domains (CNB-A and CNB-B). The multi-conformer state of Lys213 suggests that conformational selection (Changeux & Edelstein, 2011 ▸) might be important in the binding of PKA-C to PKA-R: while previous models of this region have the residue in a single conformation for either the bound or unbound states, our structure suggests that the side chain naturally samples two conformations at room temperature (Fig. 7 ▸).

We are unaware of a multi-conformer model for Asp166 having previously been suggested in the literature. Although we are not certain about the origin of this or other features of PKA that are unique to this study, it is possibly related to the room-temperature crystal growth or to data collection at 12°C. The presence of this state might be connected to the hydrolyzed ATP and the free phosphate. This model of Asp166, which serves as the catalytic base for the phosphotransfer reaction, suggests a mechanism of activity involving the water network adjacent to MG1 (Fig. 8 ▸ *b*). When Asp166 is in the A conformation, the distance between MG1 and the water O atom HOH W1A is 2.06 Å. In the B conformation this water is displaced, and MG1 is coordinated to OD2 of Asp166 with a longer distance of 3.34 Å. The change in coordination distance for MG1 going from the water O atom to the O atom of Asp166 suggests the possibility that the placement of MG1 might be less energetically favorable when Asp166 is in the B conformation compared with the A conformation. Previous studies have shown that the simultaneous release of both magnesium ions and ADP from the active site is unfeasible; instead, one magnesium (MG1) must first exit before ADP and MG2 can exit post-catalysis (Khavrutskii *et al.*, 2009 ▸; Bastidas *et al.*, 2015 ▸). The multi-conformer states for both Asp166 and associated water(s) thus might correspond to a progression of the phosphotransfer reaction and suggest a potential mechanism for this post-catalysis release.

The insights gained into PKA suggest that the MD–MX procedure may be beneficial in other crystallographic studies, producing information that cannot be obtained using other methods. (i) The MD–MX procedure yields an extended MD-derived solvent model that can be compared with that generated by water picking in crystallographic refinement. (ii) The MD solvent model and density analysis can help to guide manual adjustments when the difference density does not provide a clear picture of how to improve the structural model. (iii) It yields a picture of conformational variability that is more independent of the crystallographic data and is more integrated with the solvent model in comparison with other techniques. For example, *qFit* (Berman *et al.*, 2000 ▸; Riley *et al.*, 2021 ▸) and *phenix.ensemble_refinement* (Burnley *et al.*, 2012 ▸) generate pictures of anharmonic motions that go beyond what is possible using *B* factors, and can also yield improved *R* factors. However, *qFit* does not attempt to model solvent density and *phenix.ensemble_refinement* does not model all waters explicitly in the MD simulation step. Similarly, other non-MD-based multiple-copy refinement approaches (Kuriyan *et al.*, 1991 ▸; Pellegrini *et al.*, 1997 ▸; Wall *et al.*, 1997 ▸; Levin *et al.*, 2007 ▸) do not consider the solvent in exploring conformations. These limitations can lead to placement of side chains into nonprotein density, as we saw in the case of Lys217 (Fig. 4 ▸). In contrast, the MD–MX procedure can provide information to distinguish protein and solvent density. The MD–MX procedure also provided a clear model of multi-conformer states for residues for Lys213 and Asp166. In contrast, the information from the ensemble-refinement model was unclear for these cases: in the case of Lys213 the conformations are highly disordered, without a clear two-state distribution, while in the case of Asp166 there is limited conformational variability in the ensemble-refinement model and there is substantial difference density. It is possible that differences in the MD simulations between the methods (explicit solvent, crystalline simulation, the force field *etc.*) contribute to the difference between the MD–MX method and ensemble refinement. Even when other techniques may suggest similar remodeling ideas, the MD can provide independent information to increase or decrease confidence in these ideas.

This study provides a roadmap for using crystalline MD simulations to enhance protein crystal structures and to obtain insights into protein mechanisms; however, there are some limitations. Firstly, our MD approach currently requires restraints to the crystal structure due to present inaccuracies of the MD force field. As MD force fields improve, especially through increased comparisons with protein crystallography data, we expect to be able to address this limitation by relaxing the restraints. Secondly, our study was limited by the use of a single 2.4 Å resolution data set to seed the MD–MX procedure and an additional 1.63 Å resolution data set for validation. We do not yet know the extent to which the benefits and insights obtained using the MD–MX procedure depend on the detailed properties of these data sets. To characterize the general benefits of the MD–MX procedure, it will be necessary to apply it to a variety of systems and studies. In particular, the data used for this study were collected near room temperature, and we do not know the degree to which any advantages of the MD–MX procedure might be seen in cryocrystallography. Finally, although it is clear that the MD played a critical role in distinguishing protein from solvent and in providing suggestions about alternative conformations, further investigation will be required to determine how other elements of our procedure might have influenced the results. For example, although we used an ensemble-refinement crystal structure as a target for MD restraints, it is possible that using a single-crystal structure would yield similar results. In addition, we do not yet have a deep understanding of how much information the MD procedure ‘adds’: following the example of the development of the CC* metric (Karplus & Diederichs, 2012 ▸), by examining multiple different resolution cutoffs for data processing (and performing the whole MD–MX procedure using each resulting data set) it might be possible to characterize this rigorously.

To apply the MD–MX procedure more broadly, it will be useful to develop it into an automated workflow. Such a workflow could then be folded into the crystallographic refinement pipeline in such a way as to iteratively alter and improve the ordered water and protein structure, similar to, for example, real-space refinement or water picking and filtering. Certain aspects of the procedure lend themselves quite naturally to automation, such as preparing and performing the MD simulation and the calculation of densities. Other parts of the procedure will be more challenging to automate, such as multi-conformer modeling when there are potential water clashes.

## Supplementary Material

PDB reference: cyclic adenosine mono­phosphate-dependent protein kinase, 1.63 Å resolution, 7v0g

PDB reference: 2.4 Å resolution, 7ujx

Supplementary Figs. S1 and S2. DOI: 10.1107/S2059798322011871/lp5065sup1.pdf

## Figures and Tables

**Table 1:** Crystallographic data statistics Two 180° sweeps of the same crystal were merged using *BLEND* (Section 2); the merging statistics should be interpreted accordingly (see Foadi *et al.*, 2013 ▸). Values in parentheses are for the highest resolution bin.

	2.4 Å resolution data set (PDB entry 7ujx)	1.63 Å resolution data set (PDB entry 7v0g)
Wavelength (Å)	1.11583	1.11583
Resolution (Å)	49.53–2.40 (2.486–2.400)	37.93–1.63 (1.688–1.630)
Space group	*P*2_1_2_1_2_1_	*P*2_1_2_1_2_1_
*a*, *b*, *c* (Å)	58.976, 79.7436, 99.058	58.976, 79.7436, 99.058
α, β, γ (°)	90, 90, 90	90, 90, 90
Total reflections	245582 (25524)	271228 (26672)
Unique reflections	18878 (1866)	58660 (5770)
Multiplicity	13.0 (13.7)	4.6 (4.6)
Completeness (%)	99.94 (100.00)	99.43 (99.79)
Mean *I*/σ(*I*)	15.90 (10.39)	7.39 (1.27)
Wilson *B* (Å^2^)	21.84	19.77
*R*_merge_	0.1829 (0.7015)	0.1056 (1.374)
*R*_meas_	0.1905 (0.7294)	0.1188 (1.548)
*R*_p.i.m._	0.05272 (0.1974)	0.05338 (0.6996)
CC_1/2_	0.993 (0.959)	0.996 (0.529)
CC*	0.998 (0.989)	0.999 (0.832)
Reflections used in refinement	18876 (1867)	58659 (5763)
Reflections used for *R*_free_	1888 (187)	1999 (197)

**Table 2:** Model-refinement statistics Values in parentheses are for the highest resolution bin (see Table 1 ▸).

	PDB entry 7ujx (*R*_f_), 2.4 Å resolution	PDB entry 7v0g, 1.63 Å resolution
*R*_work_	0.1282 (0.1291)	0.1167 (0.2277)
*R*_free_	0.1765 (0.1867)	0.1626 (0.2966)
CC(work)	0.968 (0.959)	0.981 (0.820)
CC(free)	0.956 (0.880)	0.962 (0.784)
No. of non-H atoms		
Total	3547	3611
Macromolecule	3011	3076
Ligands	39	39
Solvent	497	496
Protein residues	355	354
R.m.s.d., bond lengths (Å)	0.004	0.008
R.m.s.d., angles (°)	0.79	1.00
Ramachandran statistics		
Favored (%)	97.66	98.24
Allowed (%)	2.34	1.76
Outliers (%)	0	0
Rotamer outliers (%)	1.26	0.31
Clashscore	3.8	4.68
Average *B* (Å^2^)		
Overall	29.76	30.32
Macromolecule	26.62	26.17
Ligands	22.47	23.45
Solvent	49.34	56.56

**Table 3:** *R* factors for structures refined against experimental X-ray data All rows but the last were computed using the 2.4 Å resolution data set; the last row was computed using the 1.63 Å resolution data set.

	*R*_work_	*R*_free_
Initial crystal structure (*S*)	0.1557	0.1838
Ensemble-refinement model (*E*)	0.1231	0.1726
Initial MD-revised structure (*R*_i_)	0.1328	0.1777
PDB entry 7ujx (*R*_f_)	0.1282	0.1765
PDB entry 7v0g (1.63 Å resolution data)	0.1163	0.1625
