# Supplementary material for: Molecular-dynamics simulation methods for macromolecular crystallography
Source: Acta Crystallogr D Struct Biol. 2023 Jan 1;79(Pt 1):50–65. doi: 10.1107/S2059798322011871 (PMC9815100; doi:10.1107/S2059798322011871)
Supplement: Supplementary file 1 [file d-79-00050-sup1.pdf]

STRUCTURAL
BIOLOGY

Volume 79 (2023)

Supporting information for article:

Molecular-dynamics simulation methods for macromolecular crystallography

David C. Wych, Phillip C. Aoto, Lily Vu, Alexander M. Wolff, David L. Mobley, James S. Fraser, Susan S. Taylor and Michael E. Wall

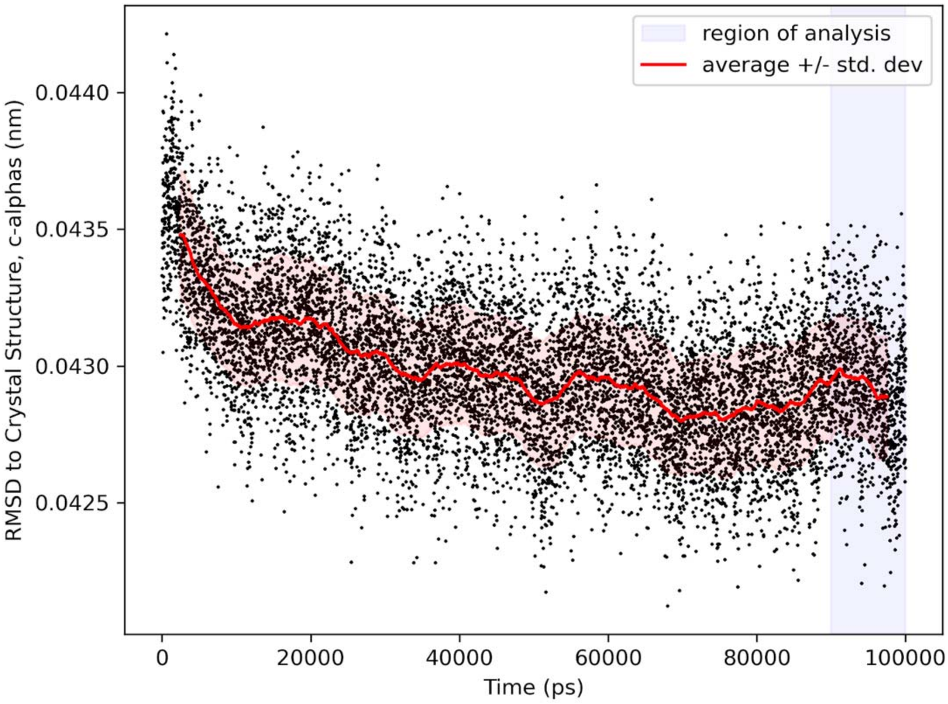

Figure S1 The c-alpha RMSD (nm) between the coordinates from the initial MD ensemble and the coordinates from production simulation. The moving average (red line) +/- standard deviation (transparent red envelope) is plotted with a window size of 5 ns (500 frames). The portion of the trajectory in which frames were processed to calculate structure factors and densities (the "region of analysis") is marked in blue.

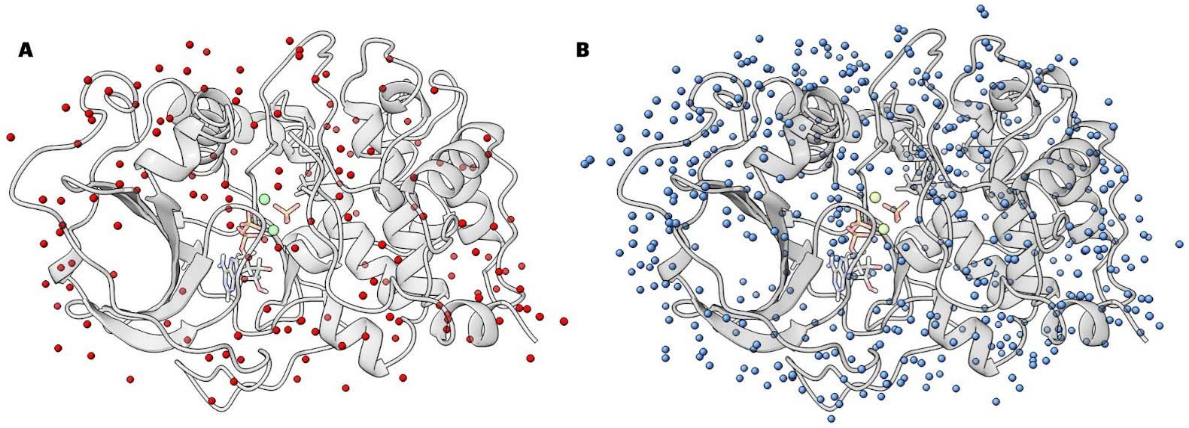

Figure S2 A comparison of the ordered water model from (A) the initial crystal structure model, *S* with 148 waters (red spheres), and (B) the MD-revised model R_f with 494 waters (blue spheres). Many of the additional waters in R_f appear in the first and second hydration layers.
